# 9-Allyl-9*H*-fluoren-9-ol

**DOI:** 10.1107/S1600536814010290

**Published:** 2014-05-17

**Authors:** Kyle S. Knight, Harvey B. Wood

**Affiliations:** a Department of Chemistry, The University of Tennessee at Chattanooga, Chattanooga, TN 37403, USA

## Abstract

The asymmetric unit of the title compound, C_16_H_14_O, contains two independent mol­ecules differing in the orientations of the allyl groups; the corresponding O—C—C(H_2_)—C(H) torsion angles are −61.01 (13) and −177.43 (10)°. In the crystal, O—H⋯O hydrogen bonds link four mol­ecules into a centrosymmetric tetra­mer, in which each hy­droxy group acts as a donor and an acceptor of hydrogen bonds.

## Related literature   

For the use of the title compound in the synthesis of spiro­cyclic ethers *via* alkene metathesis, see: Brahma *et al.* (2007 ▶).
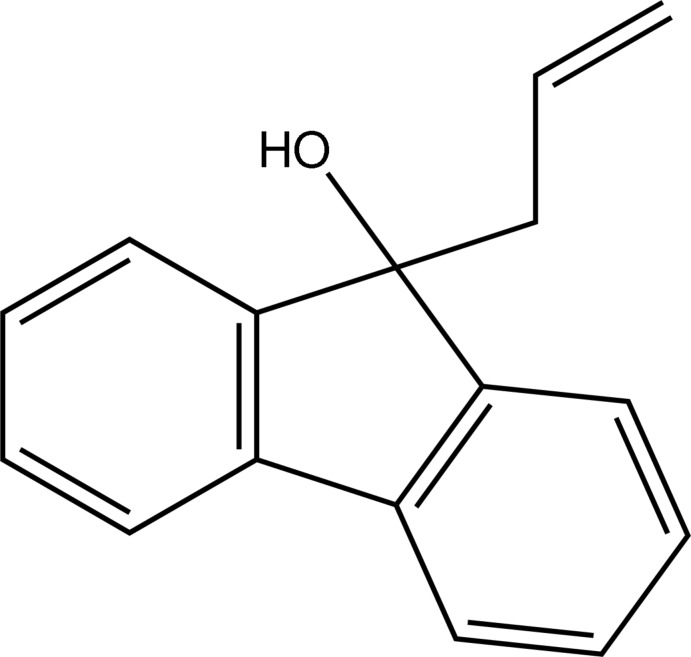


## Experimental   

### 

#### Crystal data  

C_16_H_14_O
*M* *_r_* = 222.27
Triclinic, 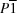

*a* = 9.3789 (15) Å
*b* = 12.2809 (18) Å
*c* = 12.936 (2) Å
α = 63.995 (4)°
β = 68.803 (4)°
γ = 69.887 (4)°
*V* = 1217.6 (3) Å^3^
*Z* = 4
Mo *K*α radiation
μ = 0.07 mm^−1^
*T* = 200 K
0.8 × 0.7 × 0.51 mm

#### Data collection  

Bruker APEXII CCD diffractometer
23092 measured reflections
4262 independent reflections
3749 reflections with *I* > 2σ(*I*)
*R*_int_ = 0.031

#### Refinement  

*R*[*F* ^2^ > 2σ(*F* ^2^)] = 0.037
*wR*(*F* ^2^) = 0.105
*S* = 1.02
4262 reflections
309 parameters
H-atom parameters constrained
Δρ_max_ = 0.16 e Å^−3^
Δρ_min_ = −0.23 e Å^−3^

### 

Data collection: *APEX2* (Bruker, 2009 ▶); cell refinement: *SAINT* (Bruker, 2009 ▶); data reduction: *SAINT*; program(s) used to solve structure: *SHELXS97* (Sheldrick, 2008 ▶); program(s) used to refine structure: *SHELXL97* (Sheldrick, 2008 ▶); molecular graphics: *OLEX2* (Dolomanov *et al.*, 2009 ▶); software used to prepare material for publication: *OLEX2*.

## Supplementary Material

Crystal structure: contains datablock(s) I. DOI: 10.1107/S1600536814010290/cv5454sup1.cif

Structure factors: contains datablock(s) I. DOI: 10.1107/S1600536814010290/cv5454Isup2.hkl

Supporting information file. DOI: 10.1107/S1600536814010290/cv5454Isup3.cdx

Supporting information file. DOI: 10.1107/S1600536814010290/cv5454Isup4.cml

CCDC reference: 1001420

Additional supporting information:  crystallographic information; 3D view; checkCIF report

## Figures and Tables

**Table 1:** Hydrogen-bond geometry (Å, °)

*D*—H⋯*A*	*D*—H	H⋯*A*	*D*⋯*A*	*D*—H⋯*A*
O1—H1⋯O2^i^	0.84	1.95	2.7709 (13)	165
O2—H2*A*⋯O1	0.84	1.93	2.7558 (15)	170

Symmetry code: (i) 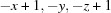.

**Fig. 1.**
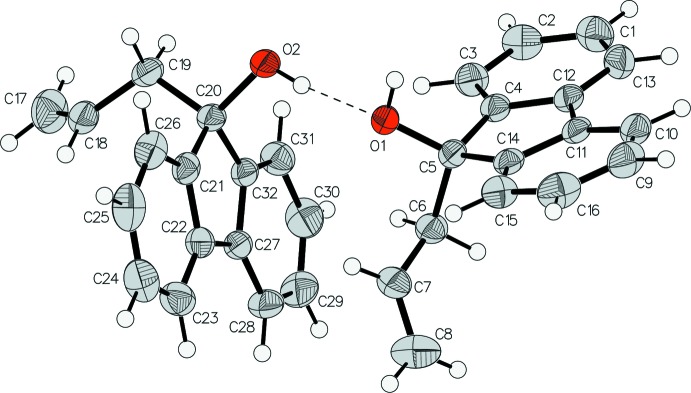
Two independent molecules of the title compound showing the atomic numbering. Displacement ellipsoids are drawn at the 50% probability level. Dashed line denotes hydrogen bond.

## supplementary crystallographic information

### 1. Comment

The homoallylic alcohol, 9-allylfluoren-9-ol, crystallizes as two symmetrically distinct molecules. The molecules are linked by a square shaped hydrogen bonding network in which symmetrically equivalent and inequivalent molecules occupy alternating positions in the corners, and each hydroxyl group acts as a donor and an acceptor to adjacent molecules. In the crystal, there is t-stacking between alternating rows of symmetrically inequivalent molecules. In one molecule the O—C—C-alkene bond, O2—C20—C19—C18, is anti with a torsion angle of -177.43 (10)° while in the other symmetrically inequivalent structure, the analogous torsion, O1—C5—C6—C7, is *gauche* and has a torsion angle of -61.01 (13)°.

### 2. Experimental

The title compound was prepared by addition of a 1.0 *M* solution of allylmagnesium chloride (0.012 mol) in tetrahydrofuran to a solution of fluorenone (0.010 mol) at 0°C. The reaction was quenched by the addition of 1.0 *M* HCl, extracted into diethyl ether and concentrated on a rotary evaporator. Suitable crystals were obtained by recrystallization from methanol.

### 3. Refinement

All H atoms bonded to C were positioned geometrically, with bond distances of 0.95 Å for C(sp2)–H and 0.95 Å for methylene, and were refined as riding, with *U*_iso_(H)= 1.2 *U*_eq_(C). H atoms bonded to O were positioned geometrically with an O–H distance of 0.84 Å, and refined as rotating, with *U*_iso_(H)= 1.5 *U*_eq_(O).

### Crystal data

**Table:**

C_16_H_14_O	*Z* = 4
*M**_r_* = 222.27	*F*(000) = 472
Triclinic, *P*1	*D*_x_ = 1.212 Mg m^−^^3^
*a* = 9.3789 (15) Å	Mo *K*α radiation, λ = 0.71073 Å
*b* = 12.2809 (18) Å	Cell parameters from 9980 reflections
*c* = 12.936 (2) Å	θ = 2.4–25.0°
α = 63.995 (4)°	µ = 0.07 mm^−^^1^
β = 68.803 (4)°	*T* = 200 K
γ = 69.887 (4)°	Prism, yellow
*V* = 1217.6 (3) Å^3^	0.8 × 0.7 × 0.51 mm

### Data collection

**Table:**

Bruker APEXII CCD diffractometer	*R*_int_ = 0.031
Graphite monochromator	θ_max_ = 25.1°, θ_min_ = 2.4°
φ and ω scans	*h* = −11→11
23092 measured reflections	*k* = −14→14
4262 independent reflections	*l* = −15→15
3749 reflections with *I* > 2σ(*I*)	

### Refinement

**Table:**

Refinement on *F*^2^	0 restraints
Least-squares matrix: full	H-atom parameters constrained
*R*[*F*^2^ > 2σ(*F*^2^)] = 0.037	*w* = 1/[σ^2^(*F*_o_^2^) + (0.063*P*)^2^ + 0.2188*P*] where *P* = (*F*_o_^2^ + 2*F*_c_^2^)/3
*wR*(*F*^2^) = 0.105	(Δ/σ)_max_ < 0.001
*S* = 1.02	Δρ_max_ = 0.16 e Å^−^^3^
4262 reflections	Δρ_min_ = −0.23 e Å^−^^3^
309 parameters	

### Special details

**Table:**

Geometry. All e.s.d.'s (except the e.s.d. in the dihedral angle between two l.s. planes) are estimated using the full covariance matrix. The cell e.s.d.'s are taken into account individually in the estimation of e.s.d.'s in distances, angles and torsion angles; correlations between e.s.d.'s in cell parameters are only used when they are defined by crystal symmetry. An approximate (isotropic) treatment of cell e.s.d.'s is used for estimating e.s.d.'s involving l.s. planes.

### Fractional atomic coordinates and isotropic or equivalent isotropic displacement parameters (Å^2^)

**Table:**

	*x*	*y*	*z*	*U*_iso_*/*U*_eq_	
O1	0.59676 (10)	0.12837 (7)	0.49408 (7)	0.0332 (2)	
H1	0.6088	0.0525	0.5364	0.050*	
O2	0.38219 (11)	0.12557 (7)	0.39598 (7)	0.0366 (2)	
H2A	0.4437	0.1363	0.4227	0.055*	
C1	0.23321 (17)	0.17419 (13)	0.88455 (12)	0.0457 (3)	
H1A	0.1578	0.1700	0.9581	0.055*	
C2	0.18390 (16)	0.20753 (13)	0.78424 (13)	0.0443 (3)	
H2	0.0751	0.2250	0.7896	0.053*	
C3	0.29179 (15)	0.21578 (12)	0.67528 (12)	0.0381 (3)	
H3	0.2577	0.2393	0.6061	0.046*	
C4	0.44978 (14)	0.18905 (11)	0.66955 (10)	0.0306 (3)	
C5	0.58817 (13)	0.19806 (10)	0.56139 (10)	0.0294 (3)	
C6	0.57498 (15)	0.33479 (11)	0.47747 (11)	0.0354 (3)	
H6A	0.4780	0.3626	0.4502	0.042*	
H6B	0.5634	0.3853	0.5234	0.042*	
C7	0.70892 (16)	0.36099 (12)	0.37113 (12)	0.0414 (3)	
H7	0.7415	0.3126	0.3227	0.050*	
C8	0.78483 (19)	0.44707 (15)	0.34030 (16)	0.0612 (4)	
H8A	0.7550	0.4969	0.3870	0.073*	
H8B	0.8696	0.4595	0.2713	0.073*	
C9	0.93625 (17)	0.04974 (13)	0.75621 (14)	0.0499 (4)	
H9	1.0097	0.0157	0.8033	0.060*	
C10	0.77762 (17)	0.07549 (12)	0.80950 (12)	0.0414 (3)	
H10	0.7417	0.0598	0.8924	0.050*	
C11	0.67245 (14)	0.12456 (10)	0.73951 (10)	0.0314 (3)	
C12	0.49977 (14)	0.15303 (10)	0.77147 (10)	0.0315 (3)	
C13	0.39132 (16)	0.14676 (12)	0.87949 (11)	0.0408 (3)	
H13	0.4248	0.1241	0.9487	0.049*	
C14	0.72606 (14)	0.14784 (10)	0.61755 (10)	0.0300 (3)	
C15	0.88448 (15)	0.12218 (12)	0.56494 (12)	0.0399 (3)	
H15	0.9209	0.1381	0.4820	0.048*	
C16	0.98985 (17)	0.07259 (14)	0.63558 (15)	0.0503 (4)	
H16	1.0993	0.0543	0.6008	0.060*	
C17	0.09971 (19)	0.33292 (15)	0.09582 (15)	0.0563 (4)	
H17A	0.0054	0.3173	0.1549	0.068*	
H17B	0.0959	0.3872	0.0168	0.068*	
C18	0.23369 (16)	0.28004 (12)	0.12297 (11)	0.0387 (3)	
H18	0.3247	0.2985	0.0609	0.046*	
C19	0.25818 (16)	0.19311 (12)	0.24235 (11)	0.0365 (3)	
H19A	0.2999	0.1078	0.2411	0.044*	
H19B	0.1554	0.1947	0.3013	0.044*	
C20	0.37019 (14)	0.22274 (10)	0.28309 (10)	0.0304 (3)	
C21	0.52751 (14)	0.23246 (11)	0.19311 (10)	0.0319 (3)	
C22	0.55426 (14)	0.35111 (11)	0.15307 (10)	0.0328 (3)	
C23	0.68727 (16)	0.38288 (14)	0.06561 (12)	0.0444 (3)	
H23	0.7056	0.4636	0.0381	0.053*	
C24	0.79322 (16)	0.29496 (16)	0.01894 (13)	0.0505 (4)	
H24	0.8843	0.3160	−0.0418	0.061*	
C25	0.76786 (16)	0.17710 (15)	0.05973 (13)	0.0502 (4)	
H25	0.8425	0.1177	0.0274	0.060*	
C26	0.63487 (15)	0.14423 (13)	0.14729 (12)	0.0420 (3)	
H26	0.6178	0.0630	0.1752	0.050*	
C27	0.42222 (14)	0.42412 (11)	0.21722 (10)	0.0316 (3)	
C28	0.39539 (17)	0.54383 (12)	0.21355 (12)	0.0433 (3)	
H28	0.4697	0.5938	0.1631	0.052*	
C29	0.25850 (19)	0.58910 (13)	0.28462 (14)	0.0511 (4)	
H29	0.2392	0.6707	0.2835	0.061*	
C30	0.14923 (18)	0.51751 (13)	0.35731 (13)	0.0499 (4)	
H30	0.0546	0.5512	0.4040	0.060*	
C31	0.17609 (15)	0.39693 (12)	0.36288 (11)	0.0398 (3)	
H31	0.1015	0.3473	0.4138	0.048*	
C32	0.31348 (14)	0.35039 (11)	0.29287 (10)	0.0298 (3)	

### Atomic displacement parameters (Å^2^)

**Table:**

	*U*^11^	*U*^22^	*U*^33^	*U*^12^	*U*^13^	*U*^23^
O1	0.0450 (5)	0.0288 (5)	0.0306 (4)	−0.0107 (4)	−0.0147 (4)	−0.0086 (3)
O2	0.0516 (5)	0.0267 (4)	0.0355 (5)	−0.0131 (4)	−0.0219 (4)	−0.0025 (4)
C1	0.0469 (8)	0.0436 (8)	0.0409 (7)	−0.0099 (6)	0.0003 (6)	−0.0192 (6)
C2	0.0339 (7)	0.0416 (8)	0.0520 (8)	−0.0060 (6)	−0.0070 (6)	−0.0166 (6)
C3	0.0372 (7)	0.0368 (7)	0.0407 (7)	−0.0071 (5)	−0.0142 (5)	−0.0115 (6)
C4	0.0360 (6)	0.0244 (6)	0.0333 (6)	−0.0073 (5)	−0.0117 (5)	−0.0092 (5)
C5	0.0341 (6)	0.0269 (6)	0.0303 (6)	−0.0078 (5)	−0.0111 (5)	−0.0096 (5)
C6	0.0402 (7)	0.0288 (6)	0.0373 (6)	−0.0076 (5)	−0.0154 (5)	−0.0073 (5)
C7	0.0493 (8)	0.0347 (7)	0.0370 (7)	−0.0125 (6)	−0.0147 (6)	−0.0046 (5)
C8	0.0552 (9)	0.0474 (9)	0.0724 (11)	−0.0217 (7)	−0.0046 (8)	−0.0154 (8)
C9	0.0513 (9)	0.0420 (8)	0.0645 (9)	−0.0094 (6)	−0.0372 (7)	−0.0088 (7)
C10	0.0557 (8)	0.0347 (7)	0.0426 (7)	−0.0143 (6)	−0.0262 (6)	−0.0072 (6)
C11	0.0421 (7)	0.0223 (6)	0.0360 (6)	−0.0098 (5)	−0.0166 (5)	−0.0085 (5)
C12	0.0408 (7)	0.0237 (6)	0.0334 (6)	−0.0084 (5)	−0.0123 (5)	−0.0100 (5)
C13	0.0537 (8)	0.0369 (7)	0.0339 (6)	−0.0102 (6)	−0.0109 (6)	−0.0145 (5)
C14	0.0359 (6)	0.0217 (6)	0.0355 (6)	−0.0075 (5)	−0.0139 (5)	−0.0081 (5)
C15	0.0378 (7)	0.0366 (7)	0.0444 (7)	−0.0091 (5)	−0.0110 (6)	−0.0124 (6)
C16	0.0353 (7)	0.0458 (8)	0.0699 (10)	−0.0060 (6)	−0.0208 (7)	−0.0169 (7)
C17	0.0571 (9)	0.0587 (10)	0.0587 (9)	−0.0055 (7)	−0.0307 (8)	−0.0186 (8)
C18	0.0449 (7)	0.0384 (7)	0.0396 (7)	−0.0118 (6)	−0.0171 (6)	−0.0129 (6)
C19	0.0447 (7)	0.0305 (7)	0.0411 (7)	−0.0135 (5)	−0.0167 (6)	−0.0101 (5)
C20	0.0377 (6)	0.0237 (6)	0.0319 (6)	−0.0080 (5)	−0.0146 (5)	−0.0061 (5)
C21	0.0348 (6)	0.0307 (6)	0.0341 (6)	−0.0043 (5)	−0.0171 (5)	−0.0106 (5)
C22	0.0349 (6)	0.0339 (7)	0.0314 (6)	−0.0093 (5)	−0.0145 (5)	−0.0071 (5)
C23	0.0425 (7)	0.0495 (8)	0.0398 (7)	−0.0178 (6)	−0.0121 (6)	−0.0075 (6)
C24	0.0342 (7)	0.0734 (11)	0.0411 (7)	−0.0111 (7)	−0.0078 (6)	−0.0200 (7)
C25	0.0389 (7)	0.0648 (10)	0.0501 (8)	0.0056 (7)	−0.0177 (6)	−0.0311 (8)
C26	0.0428 (7)	0.0400 (8)	0.0482 (8)	0.0004 (6)	−0.0197 (6)	−0.0207 (6)
C27	0.0396 (7)	0.0265 (6)	0.0317 (6)	−0.0088 (5)	−0.0165 (5)	−0.0059 (5)
C28	0.0586 (9)	0.0285 (7)	0.0457 (7)	−0.0158 (6)	−0.0196 (7)	−0.0059 (6)
C29	0.0717 (10)	0.0281 (7)	0.0567 (9)	−0.0032 (7)	−0.0239 (8)	−0.0177 (6)
C30	0.0566 (9)	0.0389 (8)	0.0492 (8)	0.0020 (7)	−0.0125 (7)	−0.0214 (7)
C31	0.0406 (7)	0.0369 (7)	0.0391 (7)	−0.0075 (6)	−0.0099 (6)	−0.0122 (6)
C32	0.0364 (6)	0.0264 (6)	0.0295 (6)	−0.0076 (5)	−0.0149 (5)	−0.0070 (5)

### Geometric parameters (Å, º)

**Table:**

O1—H1	0.8400	C15—C16	1.3906 (19)
O1—C5	1.4326 (13)	C16—H16	0.9500
O2—H2A	0.8400	C17—H17A	0.9500
O2—C20	1.4380 (14)	C17—H17B	0.9500
C1—H1A	0.9500	C17—C18	1.297 (2)
C1—C2	1.380 (2)	C18—H18	0.9500
C1—C13	1.386 (2)	C18—C19	1.4898 (17)
C2—H2	0.9500	C19—H19A	0.9900
C2—C3	1.3909 (19)	C19—H19B	0.9900
C3—H3	0.9500	C19—C20	1.5314 (16)
C3—C4	1.3843 (17)	C20—C21	1.5178 (17)
C4—C5	1.5180 (16)	C20—C32	1.5193 (16)
C4—C12	1.3952 (16)	C21—C22	1.3965 (17)
C5—C6	1.5393 (16)	C21—C26	1.3859 (18)
C5—C14	1.5227 (16)	C22—C23	1.3841 (18)
C6—H6A	0.9900	C22—C27	1.4736 (18)
C6—H6B	0.9900	C23—H23	0.9500
C6—C7	1.4880 (18)	C23—C24	1.385 (2)
C7—H7	0.9500	C24—H24	0.9500
C7—C8	1.314 (2)	C24—C25	1.381 (2)
C8—H8A	0.9500	C25—H25	0.9500
C8—H8B	0.9500	C25—C26	1.387 (2)
C9—H9	0.9500	C26—H26	0.9500
C9—C10	1.384 (2)	C27—C28	1.3856 (18)
C9—C16	1.383 (2)	C27—C32	1.3978 (17)
C10—H10	0.9500	C28—H28	0.9500
C10—C11	1.3835 (17)	C28—C29	1.381 (2)
C11—C12	1.4729 (17)	C29—H29	0.9500
C11—C14	1.3977 (17)	C29—C30	1.380 (2)
C12—C13	1.3874 (17)	C30—H30	0.9500
C13—H13	0.9500	C30—C31	1.387 (2)
C14—C15	1.3801 (18)	C31—H31	0.9500
C15—H15	0.9500	C31—C32	1.3823 (18)
			
C5—O1—H1	109.5	C15—C16—H16	119.8
C20—O2—H2A	109.5	H17A—C17—H17B	120.0
C2—C1—H1A	119.6	C18—C17—H17A	120.0
C2—C1—C13	120.87 (12)	C18—C17—H17B	120.0
C13—C1—H1A	119.6	C17—C18—H18	117.0
C1—C2—H2	119.7	C17—C18—C19	126.10 (14)
C1—C2—C3	120.68 (13)	C19—C18—H18	117.0
C3—C2—H2	119.7	C18—C19—H19A	108.7
C2—C3—H3	120.6	C18—C19—H19B	108.7
C4—C3—C2	118.72 (12)	C18—C19—C20	114.33 (10)
C4—C3—H3	120.6	H19A—C19—H19B	107.6
C3—C4—C5	128.70 (11)	C20—C19—H19A	108.7
C3—C4—C12	120.57 (11)	C20—C19—H19B	108.7
C12—C4—C5	110.67 (10)	O2—C20—C19	105.00 (9)
O1—C5—C4	113.94 (9)	O2—C20—C21	113.20 (9)
O1—C5—C6	107.01 (9)	O2—C20—C32	111.62 (9)
O1—C5—C14	112.12 (9)	C21—C20—C19	112.48 (10)
C4—C5—C6	108.96 (10)	C21—C20—C32	101.85 (9)
C4—C5—C14	101.68 (9)	C32—C20—C19	112.95 (10)
C14—C5—C6	113.19 (9)	C22—C21—C20	110.55 (10)
C5—C6—H6A	108.4	C26—C21—C20	128.87 (11)
C5—C6—H6B	108.4	C26—C21—C22	120.53 (12)
H6A—C6—H6B	107.4	C21—C22—C27	108.61 (11)
C7—C6—C5	115.68 (10)	C23—C22—C21	120.41 (12)
C7—C6—H6A	108.4	C23—C22—C27	130.97 (12)
C7—C6—H6B	108.4	C22—C23—H23	120.6
C6—C7—H7	118.1	C22—C23—C24	118.86 (14)
C8—C7—C6	123.87 (14)	C24—C23—H23	120.6
C8—C7—H7	118.1	C23—C24—H24	119.6
C7—C8—H8A	120.0	C25—C24—C23	120.71 (13)
C7—C8—H8B	120.0	C25—C24—H24	119.6
H8A—C8—H8B	120.0	C24—C25—H25	119.5
C10—C9—H9	119.4	C24—C25—C26	120.94 (13)
C16—C9—H9	119.4	C26—C25—H25	119.5
C16—C9—C10	121.18 (12)	C21—C26—C25	118.53 (13)
C9—C10—H10	120.7	C21—C26—H26	120.7
C9—C10—C11	118.59 (13)	C25—C26—H26	120.7
C11—C10—H10	120.7	C28—C27—C22	131.34 (12)
C10—C11—C12	130.85 (12)	C28—C27—C32	120.40 (12)
C10—C11—C14	120.44 (12)	C32—C27—C22	108.26 (11)
C14—C11—C12	108.59 (10)	C27—C28—H28	120.6
C4—C12—C11	108.33 (10)	C29—C28—C27	118.71 (13)
C13—C12—C4	120.36 (12)	C29—C28—H28	120.6
C13—C12—C11	131.29 (11)	C28—C29—H29	119.5
C1—C13—C12	118.78 (12)	C30—C29—C28	120.99 (13)
C1—C13—H13	120.6	C30—C29—H29	119.5
C12—C13—H13	120.6	C29—C30—H30	119.6
C11—C14—C5	110.34 (10)	C29—C30—C31	120.71 (13)
C15—C14—C5	128.98 (11)	C31—C30—H30	119.6
C15—C14—C11	120.66 (11)	C30—C31—H31	120.6
C14—C15—H15	120.6	C32—C31—C30	118.71 (13)
C14—C15—C16	118.76 (13)	C32—C31—H31	120.6
C16—C15—H15	120.6	C27—C32—C20	110.68 (10)
C9—C16—C15	120.38 (13)	C31—C32—C20	128.86 (11)
C9—C16—H16	119.8	C31—C32—C27	120.46 (11)

### Hydrogen-bond geometry (Å, º)

**Table:**

*D*—H···*A*	*D*—H	H···*A*	*D*···*A*	*D*—H···*A*
O1—H1···O2^i^	0.84	1.95	2.7709 (13)	165
O2—H2*A*···O1	0.84	1.93	2.7558 (15)	170

Symmetry code: (i) −*x*+1, −*y*, −*z*+1.
